# Releasing high positive end-expiratory pressure to a low level generates a pronounced increase in particle flow from the airways

**DOI:** 10.1186/s40635-023-00498-3

**Published:** 2023-03-17

**Authors:** Ellen Broberg, Leif Pierre, Mohammed Fakhro, Malin Malmsjö, Sandra Lindstedt, Snejana Hyllén

**Affiliations:** 1 Department of Clinical Sciences, Lund University, Lund, Sweden; 2 Department of Cardiothoracic Anaesthesia and Intensive Care, Skåne University Hospital, Entrégatan 8, Level 8, 22241 Lund, Sweden; 3 Department of Cardiothoracic Surgery, Skåne University Hospital, Lund, Sweden; 4 Department of Cardiothoracic Surgery, Rigshospitalet, Copenhagen, Denmark; 5 Department of Ophthalmology, Skåne University Hospital, Lund, Sweden; 6 Wallenberg Center for Molecular Medicine, Lund University, Lund, Sweden

**Keywords:** Mechanical ventilation, Lung function, Intensive care unit, PEEP

## Abstract

**Objectives:**

Detecting particle flow from the airways by a non-invasive analyzing technique might serve as an additional tool to monitor mechanical ventilation. In the present study, we used a customized particles in exhaled air (PExA) technique, which is an optical particle counter for the monitoring of particle flow in exhaled air. We studied particle flow while increasing and releasing positive end-expiratory pressure (PEEP). The aim of this study was to investigate the impact of different levels of PEEP on particle flow in exhaled air in an experimental setting. We hypothesized that gradually increasing PEEP will reduce the particle flow from the airways and releasing PEEP from a high level to a low level will result in increased particle flow.

**Methods:**

Five fully anesthetized domestic pigs received a gradual increase of PEEP from 5 cmH_2_O to a maximum of 25 cmH_2_O during volume-controlled ventilation. The particle count along with vital parameters and ventilator settings were collected continuously and measurements were taken after every increase in PEEP. The particle sizes measured were between 0.41 µm and 4.55 µm.

**Results:**

A significant increase in particle count was seen going from all levels of PEEP to release of PEEP. At a PEEP level of 15 cmH_2_O, there was a median particle count of 282 (154–710) compared to release of PEEP to a level of 5 cmH_2_O which led to a median particle count of 3754 (2437–10,606) (p < 0.009). A decrease in blood pressure was seen from baseline to all levels of PEEP and significantly so at a PEEP level of 20 cmH_2_O.

**Conclusions:**

In the present study, a significant increase in particle count was seen on releasing PEEP back to baseline compared to all levels of PEEP, while no changes were seen when gradually increasing PEEP. These findings further explore the significance of changes in particle flow and their part in pathophysiological processes within the lung.

## Introduction

Today, the main instruments for assessing respiration during mechanical ventilation are by monitoring pressure, volume, and airflow. The particles in exhaled air (PExA) device is an optical particle counter that detects and analyzes exhaled particles in a size between 0.41 and 4.55 μm and may add another monitoring possibility by detecting particles exhaled from the airways. Exhaled particles are believed to be produced and excreted by the respiratory lining fluid [1–3]. The PExA device has previously detected and analyzed particles of this size in spontaneously breathing humans and the particles are thought to be released during the opening and closing of the small airways. These collected particles have also been explored as potential markers for a variety of different lung diseases, such as asthma, chronic obstructive pulmonary disease and after lung transplantation in spontaneously breathing patients [4–8]. When using a customized PExA device we have previously detected and analyzed particles in exhaled air from the airways during mechanical ventilation [9–12]. We have shown that a larger tidal volume results in higher particle flow from the airways compared to smaller tidal volumes and that different ventilation modes, in our case pressure- and volume-controlled modes, are associated with different particle flow rates. Increasing blood flow through the lungs increased the particle flow and the change in blood flow was mirrored in particle flow in a stepwise manner which could be detected by PExA [9]. In another study focusing on recruitment maneuvers we showed again that the previous two different modes of ventilation display different particle flow rates [10].

In the present study, we have focused on positive end-expiratory pressure (PEEP) levels and the information that exhaled particle flow rates can give while increasing PEEP without changing either the ventilation mode or the tidal volume.

The aim of this study was to investigate the impact of various PEEP levels on particle flow rates in exhaled air in an experimental setting. We hypothesized that by increasing PEEP in a stepwise manner and thereby reducing cardiac function it will lead to a decrease in the number of exhaled particles. We also hypothesized that by mimicking an open airway being reduced, but not necessarily closed completely, by lowering PEEP from a high to a low level this will result in an increased particle flow detected by the PExA device.

## Materials and methods

### Animal preparation

Five Swedish landrace pigs with a median weight of 63 (61–65) kg were fasted overnight with free access to water. Premedication was performed with an intramuscular injection of xylazine (Rompun® vet. 20 mg/ml; Bayer AG, Leverkusen, Germany; 2 mg/kg) mixed with ketamine (Ketaminol® vet. 100 mg/ml; Farmaceutici Gellini S.p.A., Aprilia, Italy; 20 mg/kg), and a peripheral intravenous access was established in the earlobe. The pigs were then transferred to the laboratory and placed in the supine position on the operating table. Anesthesia was induced with sodium thiopental (Pentothal; Abbott Laboratories, North Chicago, Illinois, USA) and pancuronium bromide (Pavulon; N.V. Organon, Oss, The Netherlands). Anesthesia was maintained with a ketamine (Ketaminol® vet), midazolam (Midazolam Panpharma®, Oslo, Norway), and fentanyl (Leptanal®, Lilly, France) infusion. Fluid loss was compensated for by continuous infusion of Ringer’s solution. Mechanical ventilation was established with a Siemens-Elema ventilator (Servo Ventilator 300, Siemens, Solna, Sweden).

### Mechanical ventilation and gradual increase of PEEP

**Fig. 1:**
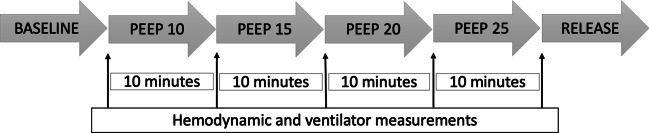
The figure shows the experimental timeline during particle in exhaled air (PExA) measurements. Increased positive end-expiratory pressure (PEEP) was recorded every 10 min. PExA measurements were performed during baseline with PEEP 5 cmH_2_O, PEEP 10 cmH_2_O, PEEP 15 cmH_2_O, PEEP 20 cmH_2_O and after release of PEEP back to PEEP 5 cmH_2_O. Hemodynamic and ventilator parameters were recorded at baseline, PEEP 10 cmH_2_O, PEEP 15 cmH_2_O, PEEP 20 cmH_2_O and after release of PEEP back to PEEP 5 cmH_2_O

All animals had an endotracheal tube of size 7.5; ventilator settings were with volume-controlled ventilation (VCV). Baseline settings were: a tidal volume of 6 mL/kg, breathing frequency 20 breaths per minute, fraction of inspired oxygen (FiO_2_) 50%, PEEP of 5 to 25 cmH_2_O, inspiratory:expiratory ratio (I:E) of 1:2 and end-inspiratory pressures of < 40 cmH_2_O were applied. These settings apart from the gradual increase of PEEP remained unchanged during the study period. PEEP was increase by 5 cmH_2_O every 10 min until end-inspiratory pressures reached a maximum of 40 cmH_2_O and/or hemodynamic instability was observed, at which time the increase in PEEP was stopped. For every pig there was a final measurement when PEEP was released back to a baseline PEEP of 5 cmH_2_O: this level was referred to as release of PEEP. The experimental timeline is presented in Fig. 1.

### PExA measurements

The PExA 2.0 instrument (PExA, Gothenburg, Sweden) provides measurements by an optical particle counter and has been described previously in conjunction with mechanical ventilation [9–11]. The instrument was connected to the outflow tract of the mechanical respiratory circuit.

The total number of particles from the airways was measured continuously by the PExA instrument and measurements were made starting at baseline, during the gradual increase of PEEP and at release of PEEP. Particles in the diameter range of 0.41–4.55 μm were measured by the PExA instrument.

### Hemodynamic and mechanical ventilation parameters

Hemodynamic and mechanical ventilation parameters were recorded at baseline, after every increase in PEEP and a final recording at release of PEEP.

### Calculations and statistics

Descriptive statistics, in the form of the number of animals, median, and the interquartile range for all parameters were analyzed. Statistical significance was tested with non-parametric tests. All statistical analyses were performed using IBM SPSS (Version 24).

## Results

### Animals

Baseline saturation was 98 (98–100)%, mean blood pressure was 92 (70–92) mmHg and pulse was 70 (45–104) beats per minute. No anatomical anomalies, signs of infection, or malignancy were found in any of the animals at autopsy. All animals reached a PEEP of 20 cmH_2_O and these results are presented in Table 1. In two animals it was not possible to produce a PEEP of 25 cmH_2_O due to the peak pressure being above 40 cmH_2_O, and three animals reached a PEEP of 25 cmH_2_O in terms of peak pressure, but were too unstable hemodynamically to be evaluated properly at this PEEP level.

**Table 1:** Mechanical ventilation data and hemodynamic parameters taken at baseline, PEEP 10, PEEP 15, PEEP 20 and at release of PEEP

	Baseline	PEEP 10	PEEP 15	PEEP 20	RELEASE
Mechanical ventilation					
Volume/minute (liter)	8 (7.8–8)	8 (7.8–8)	8 (7.8–8)	8 (7.8–8)	8 (7.8–8)
Peak pressure (cmH2O)	13.7 (13.3–14.2)	19.9 (19–20)	24.6 (21.5–26.2)	31 (28.9–32.3) *0.03*	12.4 (12–13.2)
Mean pressure (cmH2O)	7.2 (7.1–7.4)	12 (11.9–12.6)	17.1 (14.7–17.9)	22.2 (22–23.7) *0.02*	7 (6.6–7.1)
Hemodynamics					
Pulse (beats per minute)	70 (45–104)	78 (53–102)	77 (63–115)	84 (63–94)	57 (51–94)
Systolic BP (mmHg)	120 (92–135)	88 (73–140)	95 (78–118)	80 (53–92) *0.01*	77 (68–172)
Diastolic BP (mmHg)	72 (59–72)	50 (37–92)	45 (41–64)	27 (26–50)	42 (37–97)
Mean BP (mmHg)	92 (70–98)	63 (49–108)	64 (54–80)	45 (35–64) *0.01*	45 (45–107)
Saturation (%)	98 (98–100)	99 (97–100)	99 (97–100)	97 (95–100)	99 (96–100)

### Effects of mechanical ventilation on particle count from the airways during different PEEP levels

**Fig. 2:**
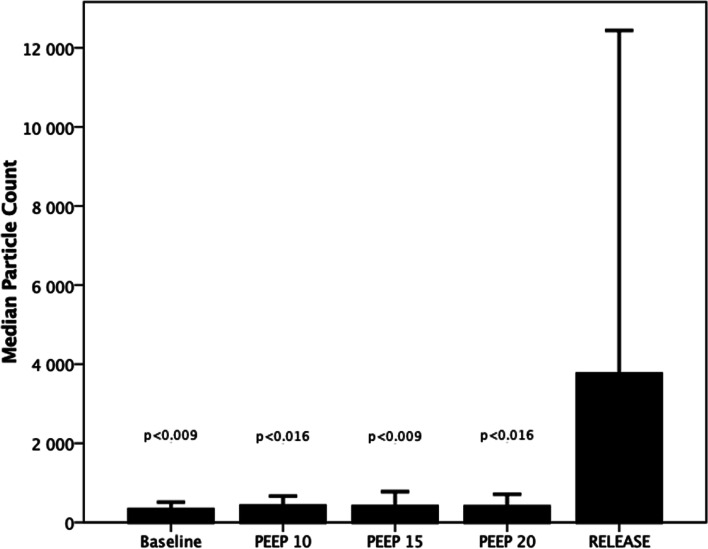
The figure shows the median particle count measured by the customized PExA during gradual increase of positive end-expiratory pressure (PEEP) when using volume-controlled ventilation. There was a statistical significance comparing release of PEEP to all levels of PEEP

Median particle count (MPC) was compared at release of PEEP to all levels of PEEP. For release of PEEP, MPC was 3754 (2437–10,606) and at baseline of PEEP, MPC was 315 (203–462) (*p* < 0.009), at PEEP 10 cmH_2_O, MPC was 523 (207–578) (*p* < 0.016), at PEEP 15 cmH_2_O, MPC was 282 (154–710) (*p* < 0.009) and at PEEP 20 cmH_2_O, MPC was 346 (176–644) (*p* < 0.016), as shown in Fig. 2. When baseline MPC was compared to MPC at the increased levels of PEEP, from 10, 15 and 20 cmH_2_O, there was no statistical significance shown at any of these levels of PEEP (*p* > 0.841).

### Mechanical ventilation and hemodynamics

Tidal volumes, ventilator pressures and hemodynamics at all measurement points during the experiment are shown in Table 1. Peak pressure and mean pressure showed a significant increase from a baseline PEEP of 5 cmH_2_O compared to a PEEP of 20 cmH_2_O. Peak pressure at baseline was 13.7 (13.3–14.2) cmH_2_O and at PEEP 20 cmH_2_O was 31 (28.9–32.3) cmH_2_O (*p* < 0.03). Mean pressure at baseline was 7.2 (7.1–7.4) cmH_2_O and at PEEP 20 cmH_2_O was 22.2 (22–23.7) cmH_2_O (*p* < 0.02).

Systolic blood pressure and mean blood pressure showed significant decreases at PEEP 20 cmH_2_O compared to the baseline PEEP of 5 cmH_2_O. Systolic blood pressure at baseline was 120 (92–135) mmHg and at PEEP 20 cmH_2_O was 80 (53–92) mmHg (*p* < 0.01). Mean blood pressure at baseline was 92 (70–98) mmHg and at PEEP 20 cmH_2_O was 45 (35–64) mmHg (*p* < 0.01).

## Discussion

We have studied the impact of PEEP on particle flow in exhaled air by gradually increasing PEEP from a baseline level of 5 cmH_2_O to 25 cmH_2_O. The particle count from the small airways stayed at a similar level during various levels of PEEP, but when PEEP was released from a high level, a significant increase in particle count from the small airways occurred and these findings were detected by the PExA device.

The MPC from the airway was similar when using PEEP 5, 10, 15 and 20 cmH_2_O, as seen in Fig. 2. Blood pressure decreased when increasing PEEP and significantly decreased at PEEP 20 cmH_2_O, but there was no similar significant decrease in MPC at PEEP 20 cm H_2_O. In a previous study with a lung animal model, we showed that by stepwise increasing blood flow through the lung there was a stepwise increase in particle count from the airways [9]. From the results in the current study, we would have expected a reduced particle count at higher levels of PEEP, especially so at a PEEP of 20 cmH_2_O since at this level blood pressure, most likely mimicking blood flow through the lung, was reduced significantly. We recorded decreasing blood pressure when increasing PEEP and this has been shown previously to be the effect that PEEP has on the cardiac function: the effect of PEEP on the cardiovascular system with reduced cardiac output has been shown in several studies [13–19]. In our study, a similar particle count from the airways was seen at both low and high levels of PEEP. One reason for this may be that the time at each PEEP level was too short to reach a steady state. It is unknown how long a time is needed to achieve a steady state for particle flow, and the time aspect in relation to PEEP and particle flow is a subject for further investigations. We believe that our findings also show the complexity of the interaction between cardiac function and lung function, where reduced blood flow through the lung for different reasons, i.e., cardiac output or changes in intrathoracic pressure, leads to different levels of particles in exhaled air. We find this of interest and further studies need to be performed.

A significant increase in MPC was seen when releasing PEEP compared to all levels of PEEP, as seen in Fig. 2. There could be several reasons why there is such an increase in MPC when releasing PEEP, such as an instant collapse of very extended airways or that the pressure induced on the production and releasing of particles does not have the same impact when increased as when decreased. A previous study with the PExA technique showed that different tidal volumes and different PEEP levels resulted in a different particle count from the airways in vivo, post-mortem and during ex vivo lung perfusion [9]. In another study, it was shown that patients on mechanical ventilation with the use of PEEP compared to normal breathing patients displayed a lower particle count [12]. These findings from these two studies indicate that an open airway during mechanical ventilation generates lower levels of particles compared to an airway that repeatedly collapses and reopens. In the present study, the increased MPC when releasing PEEP back to baseline may be a sign of open small airways being reduced but not necessarily totally collapsed. It may also show that previously closed parts of the lung, i.e., atelectasis, have been extended successfully and when PEEP is released back to baseline particles from these areas are released. The interaction between atelectasis and PEEP has been studied extensively previously and results showed that PEEP has a crucial effect on reducing atelectasis and reopening closed parts of the lung [20–24].

In all animals there were no clinically important changes in commonly used indicators for changes of lung function during mechanical ventilation such as oxygen levels or the animals’ saturation levels, but still we saw changes in the particle count when releasing PEEP. There were changes in the ventilator’s peak and mean pressures but these can be related directly to the gradual increase of PEEP.

Patients’ respiration during mechanical ventilation in intensive care is an important matter that needs constant monitoring and alteration to both improve respiration but more importantly reduce the risk of further harm. At the present time, respiration during mechanical ventilation is monitored routinely by evaluating blood gases, saturation levels and ventilator settings. In this study, we showed that the hemodynamic parameters we included, such as saturation and ventilator pressures did not change without a definite cause, but that particle count from the airways did change. Using an optical particle counter which can non-invasively analyze different particle counts from the airways has the potential to be an additional and safe way to gather further information on the impact of mechanical ventilation and, after further studies, may be an additional monitoring parameter to guide ventilation management.

## Conclusions

In the present study, we have shown that by gradually increasing PEEP, small non-significant changes in particle count were observed but, when releasing PEEP from a high level back to baseline PEEP, a significant increase in particle count was observed. Particle flow from the airways and its changes needs to be studied further in order to find the relationship between particle flow and changes in lung physiology. We hope a non-invasive technique such as the PExA can, in the future be another parameter to provide information on the optimal treatment during mechanical ventilation.

## Limitations

This study is an experimental study and has been performed under controlled conditions in a laboratory setting on five healthy animals. The number of pigs is few, and the time spent at different PEEP levels may have an impact on the particle count since the pigs’ lungs may not have had enough time to adapt to an increased PEEP.

In this study, we did not closely study the impact PEEP has on cardiac function by using for example a Swan–Ganz catheter; this measurement will be incorporated in further studies.

Despite this, these findings along with previous studies with the PExA technique have shown the complexity of lung physiology and may give rise to further understanding of the physiology of the lung and the impact of mechanical ventilation. We do believe these findings from this study can be of use when studying subjects with pre-existing lung injury.

## Data Availability

Please contact author for data requests.

## Abbreviations

I:E: Inspiratory expiratory ratio
MPC: Median particle count
PEEP: Positive end-expiratory pressure
PExA: Particles in exhaled air
VCV: Volume controlled ventilation
